# Critical Role of FoxO1 in Granulosa Cell Apoptosis Caused by Oxidative Stress and Protective Effects of Grape Seed Procyanidin B2

**DOI:** 10.1155/2016/6147345

**Published:** 2016-01-19

**Authors:** Jia-Qing Zhang, Bin-Wen Gao, Jing Wang, Qiao-Ling Ren, Jun-Feng Chen, Qiang Ma, Zi-Jing Zhang, Bao-Song Xing

**Affiliations:** Institute of Animal Husbandry and Veterinary Science, Henan Academy of Agricultural Sciences, Zhengzhou 450008, China

## Abstract

Reactive oxygen species (ROS) are closely related to the follicular granulosa cell apoptosis. Grape seed procyanidin B2 (GSPB2) has been reported to possess potent antioxidant activity. However, the GSPB2-mediated protective effects and the underlying molecular mechanisms in granulosa cell apoptosis process remain unknown. In this study, we showed for the first time that GSPB2 treatment decreased FoxO1 protein level, improved granulosa cell viability, upregulated LC3-II protein level, and reduced granulosa cell apoptosis rate. Under a condition of oxidative stress, GSPB2 reversed FoxO1 nuclear localization and increased its level in cytoplasm. In addition, FoxO1 knockdown inhibited the protective effects of GSPB2 induced. Our findings suggest that FoxO1 plays a pivotal role in regulating autophagy in granulosa cells, GSPB2 exerts a potent and beneficial role in reducing granulosa cell apoptosis and inducing autophagy process, and targeting FoxO1 could be significant in fighting against oxidative stress-reduced female reproductive system diseases.

## 1. Introduction

Ovary is the main regulator for female mammalian reproductive function, as it regulates follicle development and reproductive hormones secretion and produces mature oocytes. Numerous studies have indicated that, in female mammalian ovaries, more than 99% of developing follicles undergo atresia [1–3], which is mainly due to the apoptosis and autophagy of granulosa cells [4–6]. Reactive oxygen species (ROS), including superoxide anion radicals (O_2_^−^), hydrogen peroxide (H_2_O_2_), and hydroxyl radical (^∙^OH), are produced from normal cellular metabolism process and some external factors such as exposure to agents known to cause oxidative stress [7, 8]. Physiological levels of ROS play an important role in intracellular signal transduction, follicle development, ovulation, and gene expression [9, 10], while excessive ROS production leads to oxidative stress, which damages intracellular DNA, biomembrane lipids, proteins, and other macromolecules [11]. Accumulating evidence shows that excessive ROS cause the initiation of granulosa apoptosis and lead to antral follicle atresia [8, 12]. Furthermore, growing evidence demonstrates that high levels of ROS are associated with ovarian toxicity and result in the gradual loss of fertility [8, 13].

FoxO (Forkhead O), a subfamily of transcription factors, including FoxO1, FoxO3, FoxO4, and FoxO6, regulates diverse cellular functions such as differentiation, proliferation, metabolism, survival, and death [14]. As a key member of this family, FoxO1 plays a critical regulator role in normal development of ovarian follicles [15]. FoxO1 is highly expressed in granulosa cells of growing follicles [16] and modulates lipid and sterol biosynthesis [15]. In addition, its expression is also regulated by reproductive hormone and growth factors such as follicle-stimulating hormone (FSH) and insulin-like growth factor-I (IGF-I), which causes FoxO1 phosphorylation and promotes its nuclear exclusion via phosphatidylinositol 3-kinase (PI3K)/AKT signaling pathway [17]. Recent studies have demonstrated that FoxO1 plays an important role in the regulation of cell death caused by oxidative stress. For instance, in neurons and cardiac myocytes, FoxO1 induces cell death via the translocation from the cytoplasm to the nucleus when these cells suffered from oxidative stress [18, 19].

Diquat is a contact bipyridyl herbicide and potent prooxidant that has been widely used to induce oxidative stress in different animals and cellular models [20, 21]. Diquat can utilize molecular oxygen to produce superoxide anion radical, and subsequently hydrogen peroxide through dismutation, thus leading to serious damage to cellular components, including lipids, proteins, and nucleic acids [22].

Grape seed procyanidin extracts (GSPEs) derived from grape seeds have been reported to possess a broad spectrum of pharmacological and medicinal properties [23]. Dimeric procyanidin B2 is one of the most important components of GSPE and is probably more powerful than other polyphenols. Some studies have shown that GSPB2 exhibits protective effects against stress, inflammation, and cardiovascular diseases [24, 25]. However, there are few studies regarding the protective effects of GSPB2 on follicular granulosa cell apoptosis induced by oxidative stress. Thus, in the present study we investigated the protective effects of GSPB2 on granulosa cell apoptosis and explored the possible underlying mechanism.

## 2. Materials and Methods

### 2.1. Chemicals and Reagents

Commercially available GSPB2 powder was obtained from Solarbio Science & Technology Co., Ltd. (Beijing, China; purity ≥ 95%). Intracellular ROS red fluorescence determination kit was purchased from GENMED (Shanghai, China). Dulbecco's Modified Eagle Medium (DMEM/F-12), fetal bovine serum, penicillin, and streptomycin were purchased from GIBCO (Grand Island, NY). The* in situ* cell death fluorescein detection kit (Lot number 10770900) was obtained from Roche (Mannheim, Germany). Rabbit monoclonal anti-LC3B (#3868) antibody and mouse monoclonal anti-FoxO1 (#14952) antibody were from Cell Signaling Technology (Beverly, MA, USA). Immunohistochemical kits (SABC method) were purchased from Boshide (Wuhan, China). All other chemicals were purchased reagent grade.

### 2.2. Animals and Treatment

Female ICR mice were obtained from Experimental Animal Center of Henan Province, China. All animal experiments were performed in accordance with the recommendations in the Animal Care and Use Guidelines of the Animal Care Advisory Committee and were approved by the Animal Care Committee on the Ethics of Animal Experiments of Henan Province. All mice were acclimated for one week prior to use and maintained in a controlled environment with free access to water and food, under a 12 h light-dark cycle and at a constant temperature of 23 ± 2°C. The mice were divided randomly into four groups containing ten animals in each.  Table 1 describes the study protocols. Diquat was dissolved in normal saline and administered intraperitoneally, whereas GSPB2 were dissolved in normal saline and supplemented to mice by intragastric administration. At the end of the experiment, all mice were anesthetized and sacrificed, and ovaries were collected. Granulosa cells were collected from the left ovaries for measurement of ROS levels and other assays. The right ovaries were fixed and embedded in paraffin for apoptosis assay.

### 2.3. Cell Isolation, Culture, and Treatment

Mice were injected intraperitoneally with PMSG (10 IU) to stimulate follicular development and sacrificed 48 h later. Ovaries were obtained and transferred into Petri dishes (35 × 15 mm) filled with DMEM: F12 and then punctured with a 5-gauge needle under a surgical dissecting microscope to release granulosa cells. Next, the cells were plated at a density of 1 × 10^6^ cells/mL in six-well culture plates (2 mL per well) or 96-well culture plates (200 *μ*L per well) and cultured in DMEM/F-12 with 10% (v/v) fetal bovine serum and incubated at 37°C with 5% CO_2_. Before the formal experiment, the cultured cells were treated with a range of concentrations (from 50 *μ*M to 200 *μ*M) of H_2_O_2_ to oxidative damage and the medium was refreshed every 3 h by adding the determined concentration of H_2_O_2_. After determining cell activity, intracellular ROS levels, and apoptosis rates, the dose of 150 *μ*M H_2_O_2_ was chosen as the optimum concentration in the subsequent experiments. Likewise, various concentrations of GSPB2 (1 to 20 *μ*mol/L) were screened for the optimum concentration under H_2_O_2_-induced stress. Based on cell viability, 10 *μ*mol/L GSPB2 was determined as the optimum concentration in the later formal experiment.

### 2.4. Determination of Cell Viability

Cell viability was determined by MTT method. Briefly, the cultured granulosa cells were treated with various concentrations of H_2_O_2_ for 6 h, and the cultured cells were pretreated with different concentrations of GSPB2 for 24 h and then treatment with H_2_O_2_. To test cell viability, 200 *μ*L DMEM/F12 medium containing 0.5 mg/mL MTT was added to per well and incubated for a further 4 h. The medium was removed and replaced with 150 *μ*L of DMSO. The absorbance was measured on microplate reader at 570 nm. The percentage of living cells was calculated by the ratio of optical density of the experimental wells to that of the normal wells.

### 2.5. Measurement of Intracellular ROS

Intracellular ROS levels were measured using the GENMED intracellular ROS red fluorescence determination kit. This assay is based on the principle that, in the presence of ROS, dihydroethidium bromide (DHE) is rapidly oxidized to become highly fluorescent products. These procedures were performed according to the manufacturer's instructions. Image J software was used to analyze the optical density in each granulosa cell.

### 2.6. *In Situ* TUNEL Analysis of Apoptosis

Apoptosis of granulosa cells was detected by terminal deoxynucleotidyl transferase-mediated dUTP-biotin nick end labeling (TUNEL) assay using a kit (Roche Applied Science). The detailed procedure was performed according to the manufacturer's instructions. Laser-scanning confocal microscope (Leica) was used to obtain fluorescence images. Six fields of each coverslip were randomly selected for counting, and 100 cells were randomly counted for each field of vision. The total apoptotic cell number and the total cell number were counted for six fields of vision. The apoptosis rate was then calculated.

### 2.7. Cell Transfection and Treatments

Plasmids encoding FoxO1 shRNA or scrambled oligonucleotides were ordered from Sangon (Shanghai, China). The sequence of FoxO1 shRNA and control shRNA are given in Table 2. Knockdown of FoxO1 was performed by transfecting granulosa cells with FoxO1 shRNA. Transient transfection was performed using Lipofectamine 2000 (Invitrogen), according to the manufacturer's instructions. The transfection efficiency was confirmed by Western blot. Twenty-four hours after transfection, cultured granulosa cells were treated with 150 *μ*M H_2_O_2_ for 6 hours. Total RNA and proteins were collected and preserved at −80°C until further analysis.

### 2.8. Quantitative Real-Time Polymerase Chain Reaction (qRT-PCR)

Total RNA was extracted from granulosa cells using TRIzol reagent (Invitrogen, Carlsbad, CA, USA) according to the manufacturer protocol. First cDNA strand was synthesized using PrimeScript RT Master Mix (Takara Bio, Inc., Shiga, Japan). Quantitative real-time PCR (qRT-PCR) was conducted using a fast real-time PCR system (Roche LightCycler 480 system). Triplicate samples were assessed for each gene of interest, and GAPDH was used as a control gene. Relative expression levels were determined by the 2^−ΔΔCt^ method. Sequences of primers used for apoptosis related genes are listed in Table 3. Primer sequences for autophagy-related genes were obtained from published literatures [26, 27].

### 2.9. Western Blot Analysis

The cultured granulosa cells were lysed with ice-cold radioimmunoprecipitation assay (RIPA) buffer. The whole-cell lysates (20 mg/lane) were separated on sodium dodecyl sulfate polyacrylamide gel electrophoresis and transferred to a polyvinylidene difluoride (PVFD) membrane. After the nonspecific binding sites were blocked with 5% skim milk, the membrane was treated with anti-LC3B rabbit monoclonal antibody or anti-FoxO1 (diluted 1 : 1000) overnight at 4°C. The immunoreactive bands were demonstrated by incubation with horseradish peroxidase- (HRP-) conjugated goat anti-rabbit IgG (diluted 1 : 3000) at room temperature for 1.5 hours. Protein bands were visualized by exposing to an enhanced chemiluminescence detection system (LAS-4000 imager, Fujifilm, Tokyo, Japan). Densitometry analyses were performed and the values for target proteins were normalized to *β*-actin as the endogenous control.

### 2.10. Immunofluorescence Staining

Granulosa cells were grown on coverslips and processed following a standard protocol. Briefly, the granulosa cells were treated with 3.7% paraformaldehyde, permeabilized with 0.5% Triton X-100, and incubated with anti-FoxO1 antibody (1 : 150) for 60 min at 25°C. Then the cells were incubated with second antibody for 60 min. DAPI was used to visualize the nuclei. Laser-scanning confocal microscope (Leica) was used to obtain fluorescent images.

### 2.11. Immunohistochemistry

Paraffin-embedded whole ovarian sections were deparaffinized and rehydrated. Antigen retrieval was treated in 10 mM citric buffer. 3% H_2_O_2_ was used to reduce endogenous peroxide. Nonspecific binding was blocked with 3% bovine serum albumin for 30 minutes. After washing, sections were incubated overnight at 4°C with anti-LC3B rabbit monoclonal antibody (diluted 1 : 300), followed by incubation with a biotinylated secondary antibody for 1 hour at a dilution of 1 : 500. The sections were counterstained with hematoxylin, then dehydrated, and mounted. In the negative control group, the anti-LC3B antibody was replaced with 1% BSA.

### 2.12. Statistical Analysis

Statistical analysis was performed using the SPSS 16.0 software (SPSS Inc., Chicago, IL, USA). All values are expressed as mean ± SEM. The statistical significance between groups was analyzed by one-way ANOVA and a *P* value of < 0.05 was considered significant. All experiments were repeated at least three times.

## 3. Results

### 3.1. Effects of Diquat Alone or Combined with GSPB2 on ROS Levels in Granulosa Cells

**Figure 1:**
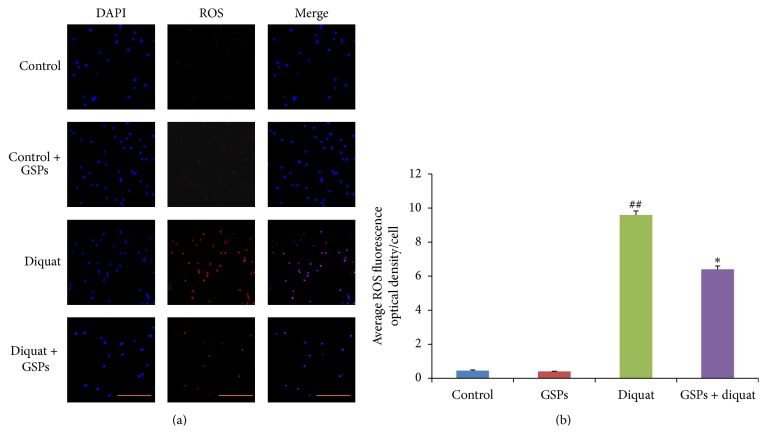
Effects of diquat alone or combined with GSPB2 on ROS levels in granulose cells. Mice were treated as described in Section 2 and granulosa cells were isolated for ROS detection. (a) The ROS levels were detected by dihydroethidium bromide fluorescence (red), and nuclei were counterstained with DAPI (blue). (b) Quantification of ROS levels in granulose cells. Results are expressed as means ± SEM, *n* = 10, ^##^ *P* < 0.05 versus control, and ^*∗*^ *P* < 0.05 versus diquat alone.

Previous studies have indicated that more than 24 mg/kg body weight of diquat in mice can result in acute toxicity [28]. The antioxidative dose for the GSPB2 was chosen because this was the most effective dose [24]. In the present study, chronic exposure to diquat (8 mg/kg, twice a week for 2 weeks) significantly induced the granulosa cell damage. The granulosa cells were collected by puncture of the dominant ovarian follicle from the left ovaries of control and treated mice. The levels of ROS in granulosa cells were examined by ROS red fluorescence determination kit. After treatment with diquat for 14 days, the ROS levels were significantly elevated in diquat group as compared to those of the control group (Figures 1(a) and 1(b)). This change was prevented by prior and concurrent supplementation of GSPB2 in diquat plus GSPB2 group. No significant differences were found between the control group and control plus GSPB2 group.

### 3.2. Effects of Diquat Alone or Combined with GSPB2 on Apoptosis in Antral Follicles

**Figure 2:**
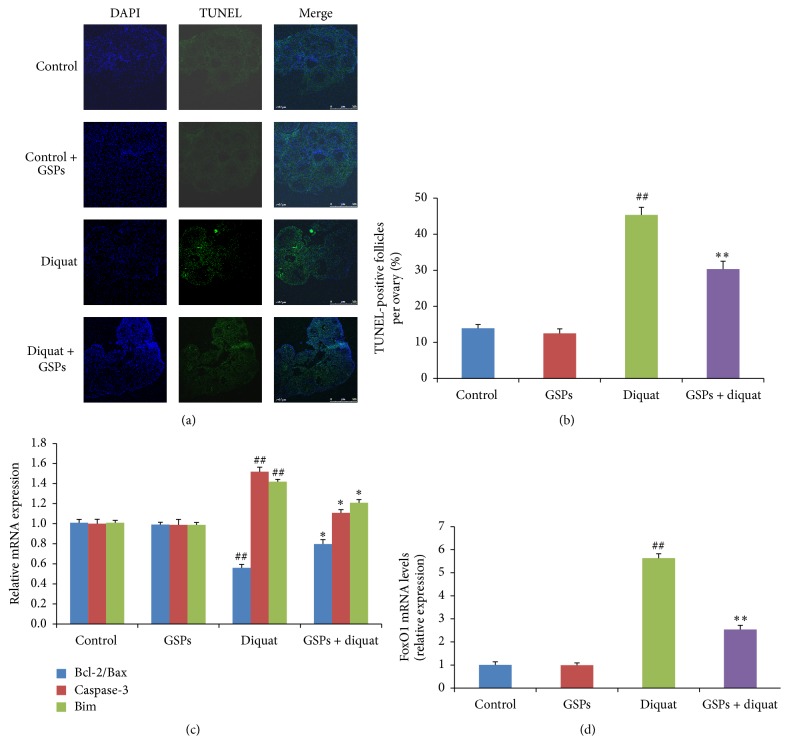
Effects of diquat alone or combined with GSPB2 on granulosa cell apoptosis in antral follicle. Mice were treated as described in Section 2 and the right ovaries were embedded in paraffin and serially sectioned for TUNEL assay. (a) Follicular granulosa cell apoptosis was assayed by TUNEL. (b) Quantification of TUNEL-positive follicles. (c) The relative expression levels of apoptosis-related genes. (d) The relative expression level of FoxO1 in granulosa cells. Results are expressed as means ± SEM, *n* = 6, ^##^ *P* < 0.05 versus control, and ^*∗*^ *P* < 0.05 versus diquat alone.

The right ovaries from control and treated animal were fixed and embedded in paraffin for the TUNEL assay. The percentage of follicular granulosa apoptosis and TUNEL-positive follicles in diquat group was higher than the control group (Figures 2(a) and 2(b)). Compared with diquat alone-treated mice, the percentage of follicular granulosa apoptosis and TUNEL-positive follicles was significantly reduced in diquat plus GSPB2 group. Quantitative PCR analysis of apoptosis-related genes showed that the expression levels of proapoptotic genes (Bim and caspase-3) and FoxO1 were significantly increased and the ratio of Bcl-2 to Bax was significantly decreased in granulosa cells from diquat group compared with the control group. Compared with diquat alone-treated mice, the expression levels of proapoptotic genes and FoxO1 were significantly decreased and the ratio of Bcl-2 to Bax was significantly increased in diquat plus GSPB2 group (Figures 2(c) and 2(d)).

### 3.3. Effects of Diquat Alone or Combined with GSPB2 on Autophagy in Antral Follicles

**Figure 3:**
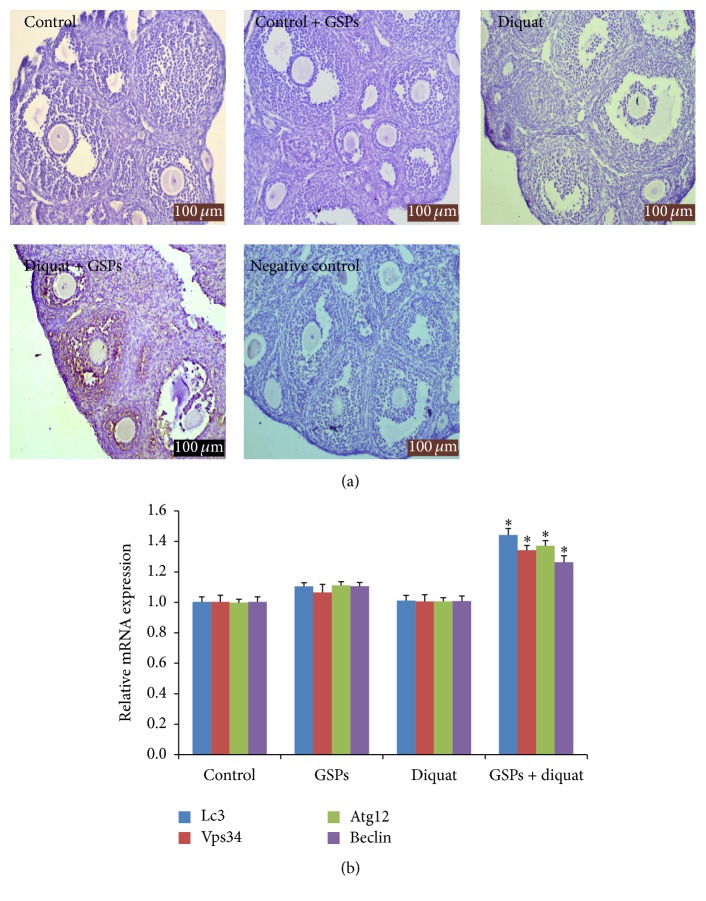
Effects of diquat alone or combined with GSPB2 on autophagy levels in antral follicles. Mice were treated with diquat or saline or in combination with GSPB2. The right ovarian sections were used for immunohistological study and granulosa cells were isolated from left ovaries to investigate the changes of autophagy-related gene expression. (a) Immunostaining of ovary sections was detected by using anti-LC3-II. (b) The relative expression levels of autophagy-related genes. Results are expressed as means ± SEM, *n* = 6, and ^*∗*^ *P* < 0.05 versus diquat alone.

Granulosa cells were collected from the left ovaries for quantitative PCR analysis. The right ovaries were fixed in 4% paraformaldehyde for immunohistochemical analysis. The LC3 protein levels were significantly enhanced in granulosa cells from diquat plus GSPB2 group compared with the diquat alone-treated group (Figure 3(a)). Consistent with the data from qRT-PCR assays, the relative mRNA expression of some key autophagy genes (Lc3, Vps34, Atg12, and Beclin) was significantly increased in granulosa cells from diquat plus GSPB2 treated mice compared with the diquat alone-treated mice (Figure 3(b)).

### 3.4. Effects of Diquat Alone or Combined with GSPB2 on Activities of Antioxidant Enzymes and MDA Content in Ovarian Tissue

**Figure 4:**
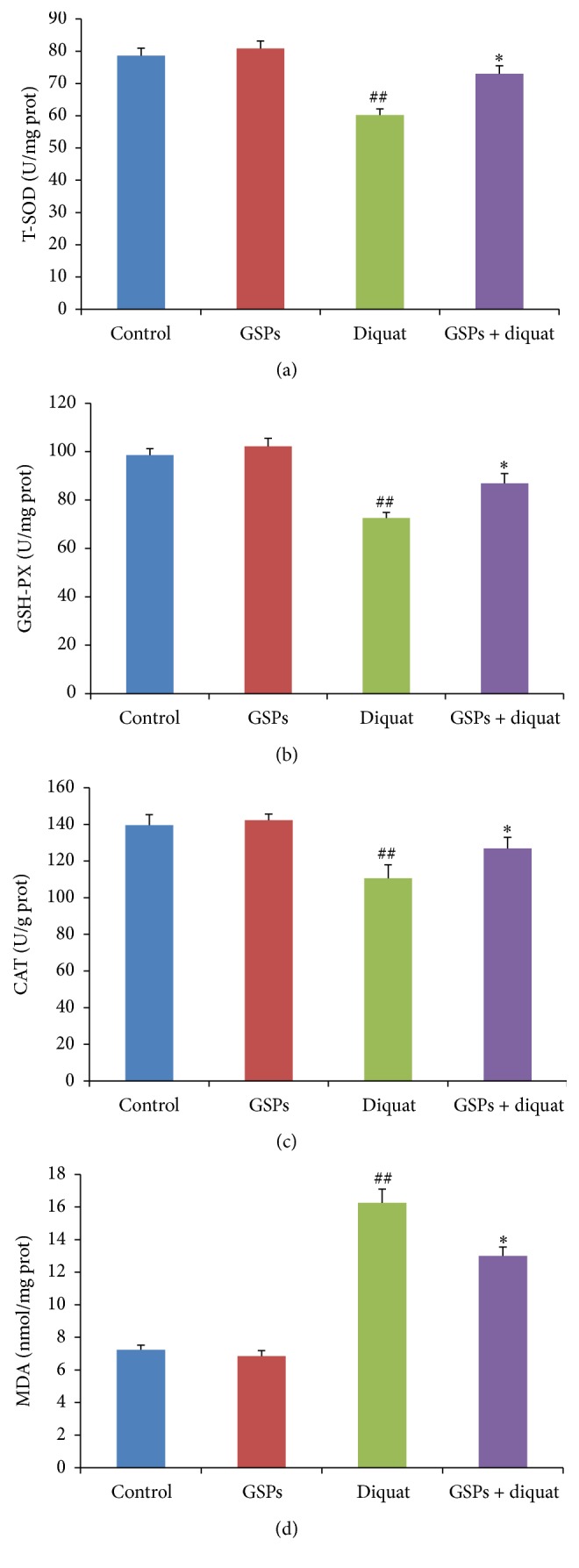
Effects of diquat alone or combined with GSPB2 on antioxidant enzyme activity and MDA content in ovarian tissue. Mice were treated with diquat or saline or in combination with GSPB2. Ovaries were then collected and homogenized for the assay of antioxidant activity. (a) T-SOD activity in ovarian tissue. (b) GSH-PX activity in ovarian tissue. (c) CAT activity in ovarian tissue. (d) MDA content in ovarian tissue. Results are expressed as means ± SEM, *n* = 6, ^##^ *P* < 0.05 versus control, and ^*∗*^ *P* < 0.05 versus diquat alone.

Ovaries from control and treated mice were collected and homogenized for the measurement of antioxidant enzymes and MDA content assay. We examined the activities of T-SOD, CAT, and GSH-Px as well as MDA content in ovarian tissues. The results showed that the activities of antioxidant enzymes (T-SOD, GPx, and CAT) were significantly decreased and the MDA contents were significantly increased in the ovary treated with diquat as compared to control group (Figures 4(a)–4(d)). This variation trend was attenuated in the ovary from mice treated with diquat plus GSPB2 as compared to diquat alone-treated mice.

### 3.5. H_2_O_2_ Induced Apoptosis in Cultured Granulosa Cells

**Figure 5:**
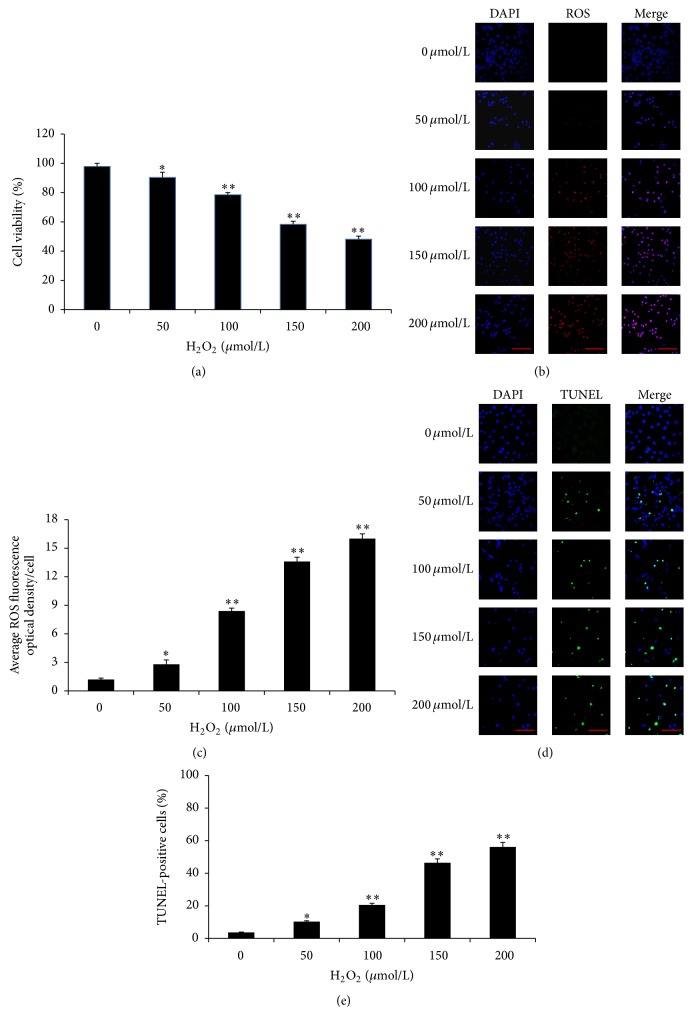
Oxidative stress-induced cytotoxicity in cultured granulosa cells. Granulosa cells collected from dominant ovarian follicle were cultured for 24 h and then exposed to different concentration of H_2_O_2_ for 6 h. (a) H_2_O_2_ significantly decreased cell survival in a dose-dependent manner. Values are expressed as the mean ± SEM, *n* = 3. ^*∗*^ *P* < 0.05, ^*∗∗*^ *P* < 0.01 H_2_O_2_ alone group versus H_2_O_2_-free group (control). (b) The ROS levels were detected by dihydroethidium bromide fluorescence (red). (c) Quantification of intracellular ROS levels. (d) Apoptosis increased in a H_2_O_2_ dose-dependent manner. (e) Quantification of the apoptosis rates. Scale bars are 100 *μ*m. Data was shown as mean ± SEM, *n* = 3. ^##^ *P* < 0.01, H_2_O_2_ alone group versus H_2_O_2_-free group (control); ^*∗∗*^ *P* < 0.01 versus H_2_O_2_ alone group.

To further investigate the protective effects of GSPB2 and underlying molecular mechanism on granulosa cells, we had primary cultured granulosa cells exposed to H_2_O_2_ and examined their effects on cell viability. Cell viability was detected after treatment with a range of concentrations (from 50 *μ*M to 200 *μ*M) of H_2_O_2_ and found that H_2_O_2_ significantly reduced cell viability in a dose-dependent manner. When the concentration of H_2_O_2_ reached 150 *μ*M, the cell viability was significantly reduced with about 50% disruption of cells as compared with that of control group (Figure 5(a)).

Moreover, treatment with 150 *μ*M H_2_O_2_ increased significantly the intracellular ROS levels (Figures 5(b) and 5(c)) and apoptosis rates in cultured cells (Figures 5(d) and 5(e)) compared with those of control group, so the dose of 150 *μ*M H_2_O_2_ was chosen as the optimal concentration for the subsequent formal trial. The results of ROS levels are shown in Figures 5(b) and 5(c); the ROS levels are increased in a dose-dependent manner when exposed to H_2_O_2_ for 6 h. To evaluate the effects of ROS accumulation on apoptosis, the cultured granulosa cells were treated with H_2_O_2_ in various concentrations as indicated for 6 h (Figures 5(d) and 5(e)). The number of TUNEL-positive cells appeared to increase dramatically and dose dependently. According to the results, 150 *μ*M H_2_O_2_ was chosen as the optimal concentration for inducing granulosa cells oxidative stress in the subsequent experiments.

### 3.6. GSPB2 Protected Granulosa Cells from H_2_O_2_-Induced Apoptosis

**Figure 6:**
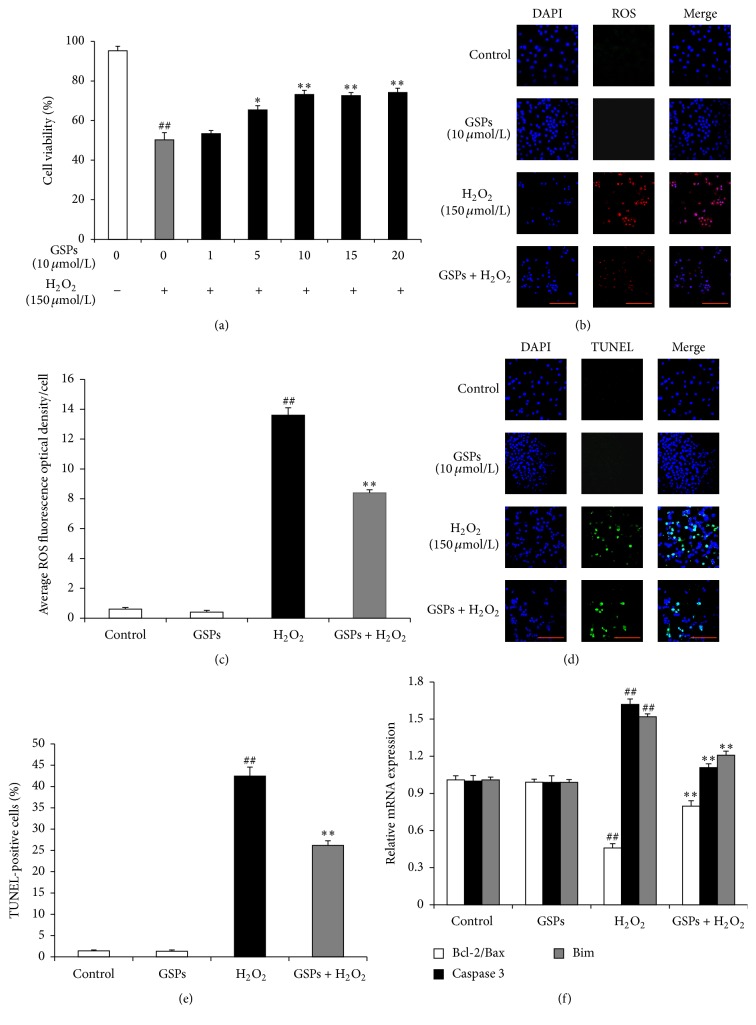
The protective effects of GSPB2 on oxidative stress-induced cytotoxicity in granulosa cells. Granulosa cells were pretreated with various concentrations of GSPB2 for 24 h and then exposed to H_2_O_2_ (150 *μ*M) for 6 h. (a) Effects of GSPB2 on the viability of granulosa cells treated with H_2_O_2_. (b) Effects of GSPB2 on the intracellular ROS levels. (c) Quantification of intracellular ROS levels. (d) Effects of GSPB2 on the apoptosis of granulosa cells. (e) Quantification of the apoptosis rates. (f) Effects of GSPB2 on the mRNA levels of apoptosis-related genes. Scale bars are 100 *μ*m. Data was shown as mean ± SEM, *n* = 3. ^##^ *P* < 0.01, H_2_O_2_ alone group versus H_2_O_2_-free group (control); ^*∗∗*^ *P* < 0.01 versus H_2_O_2_ alone group.

To evaluate whether GSPB2 protects granulosa cells from oxidative stress, we used H_2_O_2_ treatment in primary cultured cells and MTT assay was used to determine cell viability (Figure 6(a)). As illustrated, the cells treated with 150 *μ*M H_2_O_2_ for 6 h showed significant reduction in viability compared to the control group. Pretreatment with the GSPB2 (10, 15, and 30 *μ*mol/L) significantly prevented H_2_O_2_-dependent damage and increased cell viability (Figures 6(b) and 6(c)) shows intracellular ROS levels after incubation with GSPB2 (10 *μ*mol/L). The ROS levels are decreased significantly after GSPB2 treatment as compared with those of H_2_O_2_-treated group. Based on the TUNEL assay and DAPI staining, the number of TUNEL-positive granulosa cells from GSPB2-pretreated groups showed significant reduction as compared with that in H_2_O_2_-treated group (Figures 6(d) and 6(e)). qRT-PCR was performed to detect the mRNA expression levels of key apoptotic genes such as caspase-3, Bim, Bax, and Bcl-2 (Figure 6(f)). Results showed that the expression levels of caspase-3 and Bim significantly decreased, whereas the ratio of Bcl-2/Bax significantly increased in the cells pretreated with GSPB2 as compared with those in H_2_O_2_-treated group.

### 3.7. GSPB2 Promoted Autophagy in Cultured Granulosa Cells

**Figure 7:**
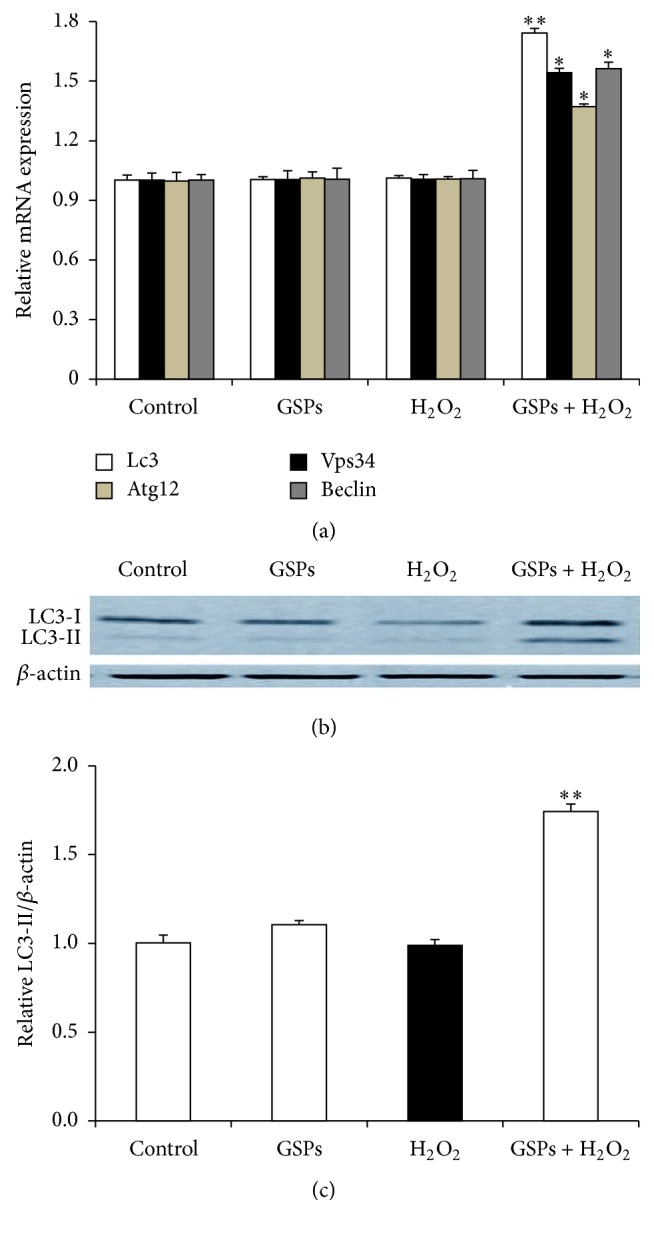
GSPB2-induced autophagy in the cultured granulosa cells. Granulosa cells were pretreated with GSPB2 (10 *μ*mol/L) for 24 h and then with H_2_O_2_ (150 *μ*M) for 6 h. (a) The relative expression levels of autophagy-related genes. (b) The induction of LC3-II expression by GSPB2 was examined using Western blotting. (c) Quantification of relative LC3-II protein levels by gradation analyses. Values are expressed as the mean ± SEM, *n* = 3, ^*∗*^ *P* < 0.05, and ^*∗∗*^ *P* < 0.01 versus H_2_O_2_ alone group.

To detect whether pretreatment with GSPB2 (10 *μ*mol/L) and exposure to 150 *μ*M H_2_O_2_ induced autophagy in cultured granulosa cells, we examined mRNA levels of key autophagy genes (Vps34, Atg12, Lc3b, and Beclin) and protein expression levels of LC3B. As shown in Figure 7(a), the mRNA levels of some key autophagy genes were increased significantly in the cells pretreated with GSPB2 as compared with those in H_2_O_2_-treated group. As predicted, the ratio of LC3-II/*β*-actin was significantly increased in the GSPB2 plus H_2_O_2_ group as compared to that in H_2_O_2_-treated group (Figures 7(b) and 7(c)). Together, these results demonstrate that autophagy is activated in the granulosa cells treated with GSPB2.

### 3.8. GSPB2 Prevented FoxO1 Expression and Nuclear Localization in Granulosa Cells

**Figure 8:**
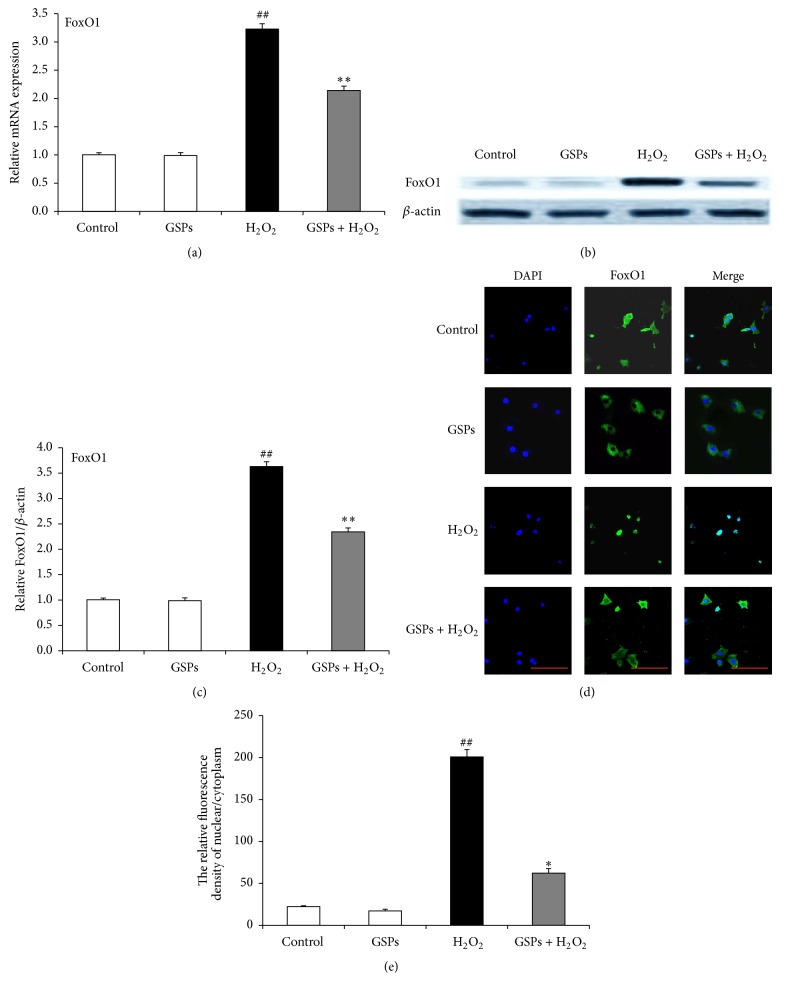
GSPB2 prevented FoxO1 expression and nuclear localization. Cultured cells were pretreated with GSPB2 (10 *μ*mol/L) for 24 h and exposed to H_2_O_2_ (150 *μ*M) for 6 h. (a) Quantitative RT-PCR was employed to detect the relative expression of FoxO1. (b) Western blotting analysis of FoxO1 protein levels in cultured granulosa cells treated with or without GSPB2. (c) Quantification of the relative FoxO1 protein level with densitometry. (d) Subcellular localization of FoxO1 in response to oxidative stress. Scale bars are 100 *μ*m. (e) The fluorescent relative quantitative analysis of nuclear/cytoplasm FoxO1. Data was shown as mean ± SEM, *n* = 3. ^##^ *P* < 0.01, H_2_O_2_ alone group versus H_2_O_2_-free group (control); ^*∗∗*^ *P* < 0.01 versus H_2_O_2_ alone group.

The effects of GSPB2 on FoxO1 expression levels in cultured granulosa cells were measured with qRT-PCR and Western blotting. The FoxO1 mRNA and protein levels were significantly elevated in the H_2_O_2_-treated granulosa cells as compared with those in control group, whereas the FoxO1 mRNA and protein levels were significantly reduced in the granulosa cells pretreated with GSPB2 compared to H_2_O_2_ alone (Figures 8(a)–8(c)). Immunofluorescence studies indicated that H_2_O_2_-induced oxidative stress leads to FoxO1 translocation from the cytoplasm to the nucleus, triggering predominant nucleus localization of FoxO1 as compared to the control group. Compared with the H_2_O_2_-induced stress group, the predominant nucleus localization of FoxO1 induced by H_2_O_2_ was suppressed in the granulosa cells pretreated with GSPB2 (Figures 8(d) and 8(e)).

### 3.9. Knockdown of FoxO1 Inhibited GSPB2-Induced Autophagy

**Figure 9:**
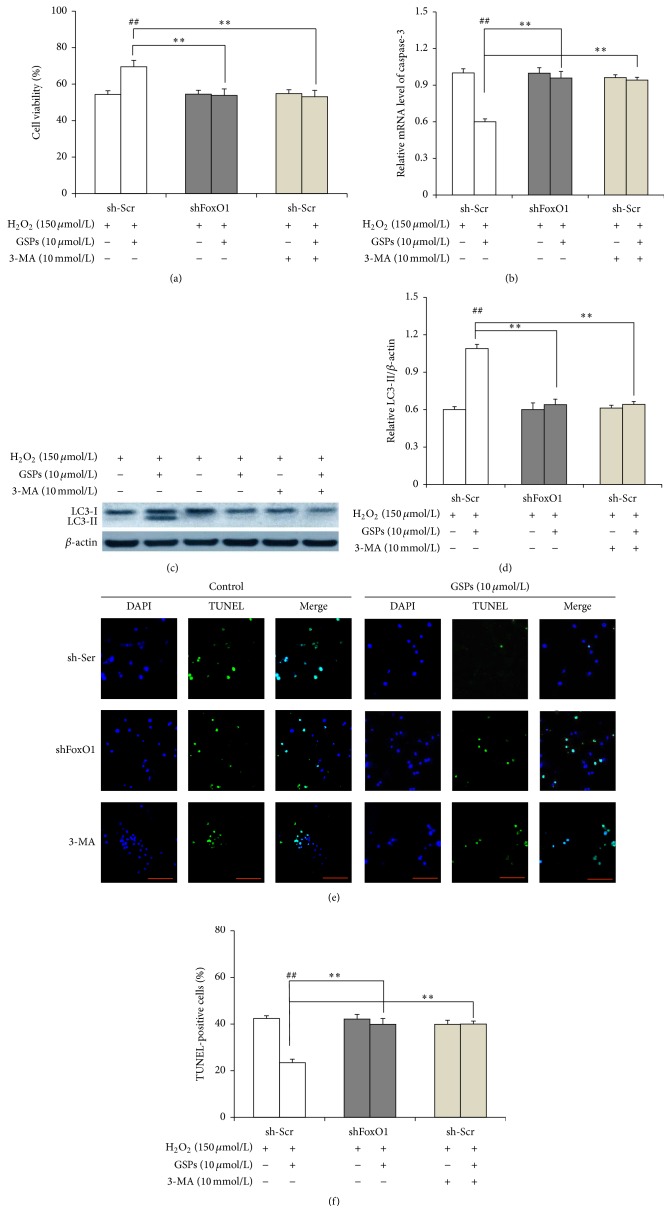
Knockdown of FoxO1 prevented GSPB2-induced autophagy. Cultured cells were transfected with FoxO1 shRNA or control scramble shRNA. Before being exposed to H_2_O_2_ (150 *μ*M) for 6 h, the cells were pretreated with GSPB2 (10 *μ*mol/L) or 3-MA (10 mmol/L) for 24 h. (a) Granulosa cell viability was tested after treatment. (b) Quantitative RT-PCR was employed to detect the relative expression of caspase-3. (c) Western blotting analysis of LC3-II protein in GSPB2-treated granulosa cells. (d) Quantification of the relative LC3-II protein level with densitometry. (e) Apoptosis was detected by a TUNEL assay. Scale bars are 100 *μ*m. (f) Quantification of TUNEL-positive cells. Values are expressed as the mean ± SEM, *n* = 3; ^##^ *P* < 0.01, sh-Scr with GSPB2 versus sh-Scr control; ^*∗∗*^ *P* < 0.01 versus sh-Scr with GSPB2 groups.

To detect whether GSPB2-induced protection is dependent on autophagy, we investigated whether the prevention of autophagy via 3-methyladenine (3-MA) or the shFoxO1 plasmid influenced GSPB2-induced protection in cultured granulosa cells. Cultured granulosa cells were pretreated with GSPB2 for 24 h and then some were transfected with FoxO1 shRNA plasmid or scrambled control shRNA plasmid for 24 h, and the others received 10 mmol/L of 3-MA, and finally exposure to 150 *μ*M H_2_O_2_ for 6 h. In shScramble (sh-Scr) group treated with GSPB2 (not with 3-MA), the cell viability was significantly recovered in H_2_O_2_-induced stress group in contrast with the control group. However, in shFoxO1 and sh-Scr treated with 3-MA groups, the same treatment did not show increased cell viability (Figure 9(a)). To further verify this result, we determined the expression levels of caspase-3 mRNA and LC3B protein in these groups. The expression level of caspase-3 significantly increased in the shFoxO1 and 3-MA groups (Figure 9(b)). Furthermore, GSPB2 induced the improvement of LC3-II protein in the cells transfected with sh-Scr vector, whereas the other groups did not increase in this protein level (Figures 9(c) and 9(d)). We next evaluated the effects of FoxO1 knockdown or 3-MA supplement on apoptosis in H_2_O_2_-induced stress process. TUNEL staining results indicated that shFoxO1 and 3-MA groups had a significant increase in apoptosis rates, regardless of the supplement of GSPB2 (Figures 9(e) and 9(f)). Together, these results show that inhibiting autophagic process or FoxO1 knockdown in granulosa cells can prevent the protective effects of GSPB2.

## 4. Discussion

Oxidative stress affects multiple female reproductive processes from ovarian follicular development to oocyte maturation, fertilization, embryo development, and pregnancy [13]. In addition, numerous studies have shown that oxidative stress plays a central role in physiological and pathological processes of infertility and assisted fertility, normal cycling ovaries, and cyclical endometrial changes [29].* In vitro* and* in vivo* supplementation of antioxidants is a strategy for overcoming oxidative stress and enhancing female fertility [13, 30]. GSPB2 is one of the main components of procyanidin extracts and has been reported to possess more potent antioxidant and anti-inflammation properties greater than B1, B4, and B5 [31]. Recent studies have shown that GSPB2 can prevent AGEs (advanced glycation end products) induced ROS generation and inhibit the human umbilical vein endothelial cell (HUVEC) apoptosis [32, 33]. In the present study, we investigated the effect of GSPB2 on H_2_O_2_-induced granulosa cell apoptosis and found that GSPB2 significantly inhibited the cultured granulosa cell apoptosis by downregulating FoxO1 expression in mRNA and protein levels. Moreover, GSPB2 significantly reduced intracellular ROS production in H_2_O_2_-treated cells. The reduction in ROS production might be related to a more direct role of the GSPB2 in the rescue of granulosa cell apoptosis induced by oxidative stress. In our present* in vivo* and* in vitro* studies, for the first time, we found that GSPB2 protected follicular granulosa cell survival from oxidative stress via triggering autophagic process. As previously reported, basal levels of autophagy are important in maintaining cellular homeostasis by efficient removal and recycling of damaged organelles and protein aggregates [34]. Our results showed that GSPB2 supplementation enhanced antioxidant ability and decreased oxidative stress and that GSPB2 reduced apoptosis and increased autophagy caused by H_2_O_2_-induced stress.

In female mammalian ovaries, more than 99% of the growing follicles undergo atresia and degeneration during follicular growth and development [1]. Recent studies have suggested that apoptosis and autophagy are involved in the regulation of granulosa cell death during ovarian follicular development and atresia [6, 35]. Furthermore, numerous studies have demonstrated that autophagy can be triggered by various stimuli that induce apoptosis, particularly oxidative stress [36, 37]. Many animal and human studies have demonstrated that ROS exist in the female reproductive tract, including ovaries and embryos [8, 38]. ROS are involved in the modulation of multiple physiological reproductive functions such as oocyte maturation, ovarian steroidogenesis, female fertility disorders, and the age-related decline in fertility [39]. Diquat has been widely used as a contact herbicide for the control of aquatic weeds and broad-leaved weeds among fruit, vegetables, and other crops [40]. Diquat is a redox cycling agent that generates ROS in cells. Thus, in this study, we investigated the toxic effects of diquat on ovarian oxidative damage and the protective role of GSPB2 against diquat-induced oxidative stress in ovaries. Our results showed that ROS levels are significantly increased in follicular granulosa cells (Figures 1(a) and 1(b)) and ovarian tissues of mice exposed to diquat. Furthermore, significant increases in granulosa cell apoptosis and TUNEL-positive follicles were also observed after diquat treatment (Figures 2(a) and 2(b)). Our results suggested that chronic exposure to diquat had toxic effects on the ovaries via enhancing ROS production and inducing oxidative damage and that GSPB2 treatment enhanced antioxidant ability and reduced oxidative stress.

In the female mammalian ovary, FoxO1 is highly expressed in follicular granulosa cells of growing follicles [16]. Majority of granulosa cells become apoptotic when growing follicles undergo atresia and degeneration, and FoxO1 has been reported to play a pivotal role in this process [15, 41]. FoxO1 is actively involved in the process of proapoptosis via a mitochondria-independent and mitochondria-dependent manner [42]. Moreover, FoxO1 activity can be regulated by* in vivo* and* in vitro* growth factors and stress factors [43]. In response to growth factors such as FSH, IGF-1, glucose, and insulin, protein kinase B (Akt) and extracellular signal-regulated kinase (ERK) can phosphorylate FoxO1, inducing FoxO1 interaction with chaperone proteins 14-3-3 and causing its cytoplasmic retention [41, 44, 45]. In response to oxidative stress, c-Jun N-terminal protein kinases (JNK), known as stress-activated protein kinases, are activated by oxidative stress [46, 47]. Activated JNK may phosphorylate 14-3-3 and interfere with 14-3-3/FoxO1 binding, leading to FoxO1 release from its interaction with 14-3-3 and promoting its nuclear translocation, thereby resulting in proapoptotic signaling via induction of the transcription of proapoptotic target genes [48–50]. Our results showed that, after treatment with 150 *μ*M H_2_O_2_, the FoxO1 was localized to the nuclei. Moreover, GSPB2 supplement partly reverses this change via increasing the FoxO1 level in the cytoplasm (Figures 8(d) and 8(e)) and improves cell viability by inhibiting apoptosis and inducing autophagic process. Furthermore, GSPB2 induced a beneficial autophagic process that was prevented when shFoxO1 was transfected to cultured granulosa cells, with the same result as the group exposed to 3-MA. The GSPB2-induced autophagic process was inhibited in shFoxO1 and 3-MA groups. Furthermore, shFoxO1 or 3-MA improved caspase-3 expression and reduced LC3-II protein level (Figures 9(a)–9(e)).

In summary, this study, for the first time, indicates the potential protective activity of GSPB2 from procyanidin extracts on oxidative stress-induced granulosa cell apoptosis and also clarifies the potential underlying molecular mechanism. Moreover, these findings give us a clue that targeting key transcription factor may be a potential therapeutic avenue for the treatment of oxidative stress-related female reproductive system diseases.

## Figures and Tables

**Table 1:** Scheme of the experiment to study the protective role of GSPB2 in diquat-induced oxidative damage.

Group	Treated with	Treatment time
Control (I)	Saline (orally, once daily)	21 days

Control + GSPB2 (II)	GSPB2 (30 mg/kg, orally, once daily)	21 days

Diquat (III)	Saline (as in group I) + diquat (8 mg/kg, i.p. twice weekly)	Saline treatment for 7 days and then treatment with diquat for two weeks

Diquat + GSPB2 (IV)	GSPB2 (as in group II) + diquat (as in group III)	GSPB2 treatment for 21 days, on day 7, treated with diquat for two weeks

**Table 2:** shFoxO1 and control scrambled sequence.

shFoxO1 sequence	A: 5′-CACCGCAGCCAGGCATCTCATAACATTCAAGAGATGTTATGAGATGCCTGGCTGCTTTTTTG-3′
	S: 5′-GATCCAAAAAAGCAGCCAGGCATCTCATAACATCTCTTGAATGTTATGAGATGCCTGGCTGC-3′

Control sequence	A: 5′-CACCGTTCTCCGAACGTGTCACGTCAAGAGATTACGTGACACGTTCGGAGAATTTTTTG-3′
	S: 5′-GATCCAAAAAATTCTCCGAACGTGTCACGTAATCTCTTGACGTGACACGTTCGGAGAAC-3′

**Table 3:** Primer sequences for RT-PCR.

Gene	Accession number	Primer sequences (5′-3′)	Product size (bp)	Annealing temperature (°C)
GAPDH	NM_008084.2	F: ATGGTGAAGGTCGGTGTGAACG R: CTCGCTCCTGGAAGATGGTGATG	452	56

Bax	NM_007527.3	F: CCAGGATGCGTCCACCAAGA R: GGTGAGGACTCCAGCCACAA	394	57

Bcl-2	NM_009741.3	F: GTGGATGACTGAGTACCTGAACC R: AGCCAGGAGAAATCAAACAGAG	120	60

Bim	NM_009754.3	F: TATGGAGAAGGCATTGAC R: TGTGGTGATGAACAGAGG	207	56

Caspase-3	NM_009810.2	F: ACAGCACCTGGTTACTATTC R: CAGTTCTTTCGTGAGCAT	255	54
